# A novel phosphatidylinositol 3-kinase (PI3K) inhibitor directs a potent FOXO-dependent, p53-independent cell cycle arrest phenotype characterized by the differential induction of a subset of FOXO-regulated genes

**DOI:** 10.1186/s13058-014-0482-y

**Published:** 2014-12-09

**Authors:** Richard Hill, Ravi Kiran Reddy Kalathur, Sergio Callejas, Laura Colaço, Ricardo Brandão, Beatriz Serelde, Antonio Cebriá, Carmen Blanco-Aparicio, Joaquín Pastor, Matthias Futschik, Ana Dopazo, Wolfgang Link

**Affiliations:** 1 IBB-Institute for Biotechnology and Bioengineering, Centre for Molecular and Structural Biomedicine (CBME), University of Algarve, Campus de Gambelas, Faro, 8005-139 Portugal; 2 Regenerative Medicine Program, Department of Biomedical Sciences and Medicine, University of Algarve, Campus de Gambelas, Faro, 8005-139 Portugal; 3 Genomics Unit, Centro Nacional de Investigaciones Cardiovasculares (CNIC), Calle de Melchor Fernández Almagro 3, Madrid, 28029 Spain; 4 Experimental Therapeutics Program, Spanish National Cancer Research Centre (CNIO), Calle de Melchor Fernández Almagro 3, Madrid, 28029 Spain

## Abstract

**Introduction:**

The activation of the phosphoinositide 3-kinase (PI3K)/AKT signalling pathway is one the most frequent genetic events in breast cancer, consequently the development of PI3K inhibitors has attracted much attention. Here we evaluate the effect of PI3K inhibition on global gene expression in breast cancer cells.

**Methods:**

We used a range of methodologies that include *in silico* compound analysis, *in vitro* kinase assays, cell invasion assays, proliferation assays, genome-wide transcription studies (Agilent Technologies full genome arrays), gene set enrichment analysis, quantitative real-time PCR, immunoblotting in addition to chromatin immunoprecipitation.

**Results:**

We defined the physico-chemical and the biological properties of ETP-45658, a novel potent PI3K inhibitor. We demonstrated that ETP-45658 potently inhibited cell proliferation within a broad range of human cancer cells, most potently suppressing the growth of breast cancer cells via inhibiting cell cycle. We show that this response is Forkhead box O (FOXO) protein dependent and p53 independent. Our genome-wide microarray analysis revealed that the cell cycle was the most affected biological process after exposure to ETP-45658 (or our control PI3K inhibitor PI-103), that despite the multiple transcription factors that are regulated by the PI3K/AKT signalling cascade, only the binding sites for FOXO transcription factors were significantly enriched and only a subset of all FOXO-dependent genes were induced. This disparity in gene transcription was not due to differential FOXO promoter recruitment.

**Conclusions:**

The constitutive activation of PI3Ks and thus the exclusion of FOXO transcription factors from the nucleus is a key feature of breast cancer. Our results presented here highlight that PI3K inhibition activates specific FOXO-dependent genes that mediate cell cycle arrest in breast cancer cells.

**Electronic supplementary material:**

The online version of this article (doi:10.1186/s13058-014-0482-y) contains supplementary material, which is available to authorized users.

## Introduction

Breast cancer is the most common and the leading cause of cancer deaths in women [1]. Similar to other cancers, breast cancers are extremely heterogeneous with significant attention directed towards screening and targeting the epidermal growth factor HER2 and the estrogen receptor alpha. However, in addition to these two molecular targets, an extremely high percentage of breast cancers are characterized by the constitutive activation of phosphatidylinositol 3-kinases (PI3Ks) [2].

PI3Ks are a family of lipid and protein kinases that are divided into three classes based on their primary structure and *in vitro* substrate specificity [3]-[5]. The best studied class I PI3Ks are heterodimeric kinases that are composed of a catalytic subunit and a regulatory adaptor protein. The activation of PI3Ks can be triggered by growth factors and insulin that target the PI3K catalytic subunit to the plasma membrane placing it in close proximity with its substrate phosphatidylinositol 4,5-bisphosphate (PIP2) [6]. The class I PI3Ks preferentially phosphorylate PIP2 to generate phosphatidylinositol 3,4,5-trisphosphate (PIP3) that as a secondary messenger activates the serine/threonine kinase AKT [7]. The PI3K signalling cascade is controlled by the dual lipid and protein phosphatase PTEN that negatively regulates the intracellular levels of PIP3 [8]. The constitutive activation of the PI3K/AKT signalling cascade is very common in cancer and occurs at different levels typically either activating mutations in the genes encoding the kinases PI3K (*PIK3CA*) or AKT (*AKT1*) or the reduced expression or ablation of the phosphotase PTEN [9]-[12]. Furthermore the deregulation of the PI3K/AKT signalling cascade has been implicated in the deregulation of almost all the aspects of cell physiology that promotes cell transformation including cell cycle progression, enhanced chemotherapeutic resistance, elevated cell metabolism, increased resistance to hypoxia and tumour metastasis [12],[13]. Many of these processes are controlled by the forkhead (FOXO) transcription family of proteins that bind to a conserved DNA motif (TTGTTTAC) driving transcription of crucial effecter proteins [14],[15]. The FOXO transcription factors are directly phosphorylated by AKT that promotes their export from the nucleus-abolishing FOXO-dependent gene transcription, thus ensuring that FOXO activity is suppressed [16]. Given the importance of PI3K signalling in breast cancer and the overwhelming degree of validation for PI3K as a therapeutic target, it is not surprising that the pharmacological inhibition of PI3Ks are considered to be among the most promising strategies in drug development for cancer therapy [11]. Consequently a variety of small molecules with different mechanisms of action (including pan-PI3K, dual PI3K/mTOR, and isoform-specific PI3K inhibitors) have been developed and entered a range of clinical trials [5].

We have recently identified pyrazolopyrimidine derivatives as biochemical inhibitors of PI3K and based on these results, we developed the potent and selective lead compound ETP-45658 [17]. Here we report our detailed comparative analysis of the chemical, physical and biological properties of ETP-45658 and the reference PI3K inhibitor PI-103 in a broad range of cancer cells particularly breast cancer cells. Furthermore we evaluated and validated the genome-wide transcriptional changes in breast cancer cells following exposure to ETP45658 or PI-103 and demonstrate elucidating the differential induction of specific FOXO-regulated genes.

## Material and methods

### Cell culture and compounds

Cell lines were obtained from the American Type Culture Collection (ATTC). U2OS were cultured in Dulbecco’s modified Eagle’s medium. PC3, MCF7, HCT116, MV4.11 and NCIH460 were grown in RPMI. All media were supplemented with 10% foetal bovine serum (Sigma-Aldrich, St Louis, MO, USA) and antibiotics-antimycotics. The procedures for the synthesis of ETP-45658 and PI-103 have been described previously [17].

### Kinase assays

The kinase activity of PI3Kα, PI3Kβ, PI3Kδ and PI3Kγ, mutant PI3Kα (H1047R and E542K) mammalian target of rapamycin (mTOR) and DNA-PK was measured as described previously [17]. The protein kinase assays depicted in Figure S1 in Additional file 1 and Table S2 in Additional file 2 were performed at ProQinase, GmbH [18].

### *In silico*assessment of physiochemical properties

To calculate the *in silico* parameters of each compound, we analysed both agents using PhysChem Batch software (ACD20, Advanced Chemistry Development, Inc. Version 12 ACD/Labs, Toronto, ON, Canada) that is based on the quantitative structure-property relationship (QSPR) methodology (including pKa and LogD values).

### Boyden chamber matrigel invasion assay

Cell-invasive capacity was determined using two-compartment Boyden chamber matrigel invasion assay (BD BioCoat™ Matrigel™ Invasion Chambers, BD Biosciences 354480, BD Biosciences, San Jose, CA, USA). MDA-MB231 cells were allowed to invade for 72 hours at 37°C. All of the inserts and the liquid in each well of the companion plate were removed. 10% serum RPMI medium with 5 μM calcein was added to each and incubated for 1 hour (in darkness). Fluorescence was measured with a luminometer (Envision, PerkinElmer, Inc, Waltham, MA, USA) at 485/535 nm and the results were analyzed in Activity Base (IDBS, Guildford, UK).

### Proliferation assays

Cells were seeded in 96-well microtitre plates. Compounds (in DMSO) were added to each well (at a final concentration of 10 μM). The medium was removed from the cells and replaced with 0.2 ml of medium containing either compound for 72 hours and then processed for MTT assay (Promega Corp, Madison, WI, USA) N = 6.

### Cell cycle analysis

The effect on the cell cycle following treatment with each compound was assessed by flow cytometry. Cells were grown to 70% confluence prior to drug treatment (1 to 10 μM) for 24 hours. Cells were stained with 10 μl of propidium iodide (Sigma-Aldrich). A total of 20,000 size gated cells were analysed by FACSCalibur (BD Biosciences).

### Immunoblotting

Sub-confluent cells were incubated under the conditions indicated in each figure and washed twice with phosphate-buffered saline (PBS) prior to lysis. RIPA lysis buffer was added (50 mM TrisHCl, 150 mM NaCl, 1% NP-40, 2 mM Na_3_VO_4_, 100 mM NaF, 20 mM Na_4_P_2_O_7_ and 1x protease inhibitor cocktail (Roche Molecular Biochemicals, Indianapolis, IN, USA). The membranes were incubated overnight for total AKT, phospho-serine-473-AKT, total p53 (DO1) (Santa Cruz Biotechnology, Dallas, TX, USA), phospho-threonine 32-FOXO3a (Merck Millipore, Darmstadt, Germany) and α-tubulin (Sigma-Aldrich). Secondary antibodies used were anti-mouse, goat or rabbit immunoglobulin G (IgG)-horseradish peroxidase (HRP) (Santa Cruz Biotechnology) as appropriate. Visualisation of bands was achieved using ChemiDoc imaging system (BioRad Laboratories Inc, Hercules, CA, USA) after membrane treatment with Ecl + (Amersham, Little Chalfont, UK).

### Microarray gene expression analysis: RNA amplification and labelling

Total RNA was prepared from cell lysates using the RNeasy total RNA Mini kit (Qiagen, Courtaboeuf, France), according to the manufacturer’s protocol. The amount and purity of total RNA was determined (NanoDrop, Thermo Fisher Scientific, Waltham, MA, USA) and the integrity of the RNA was assessed using a 2100 Bioanalyzer (Agilent Technologies, Santa Clara, CA, USA). One-Colour Microarray-Based Gene Expression Analysis Protocol (Agilent Technologies) was used to amplify and label RNA. Samples were hybridised to whole human genome microarray 4 x 44K (G4112F, Agilent Technologies). For each condition (one control (DMSO) and two treatments (ETP-45658 and PI-103), four replicate hybridisations were carried out. Arrays were scanned at 5 μm resolution on an Agilent DNA Microarray Scanner (G2565BA, Agilent Technologies). Microarray data has been deposited at Gene Expression Omnibus (GEO) (accession number GSE56579).

### Microarray data analysis

Data extraction from each array image was carried out using extraction software provided by Agilent Technologies. Data analysis was performed in R [19] using various Bioconductor packages. The background-corrected data was normalised using the quantile normalisation method [20]. To identify differences in gene expression between treated and control samples, the linear models for microarray data (limma) package [21] was used. *P* values were adjusted by utilisation of the Benjamini-Hochberg (BH) correction methodology [22]. Genes with an adjusted *P* value (that is those with a false discovery rate (FDR) of ≤0.05 and an absolute fold change of ≥1.5) were considered differentially expressed.

### Functional enrichment analysis

Two complementary enrichment analysis tools were applied to our data: standard enrichment analysis (SEA) [23] of differentially expressed genes and gene set enrichment analysis (GSEA) [24]. SEA for biological processes (defined in the Gene Ontology database) [25] or pathways (defined in the KEGG database) [26] as a control reference was conducted in R using several publically available Bioconductor resources. The significance of each biological processes or pathways identified was calculated using the hypergeometric test (equivalent to Fisher’s exact test). Complementarily, we carried out GSEA to evaluate if curated gene sets show statistically significant and concordant differential expression between given conditions [24]. The curated gene sets used in our GSEA analysis were obtained from the molecular signature database (MSigDB) [27]. *P* values from our SEA and GSEA were adjusted for multiple testing and converted to FDR using the BH procedure for SEA or the method implemented in the GSEA package, respectively [22],[24].

### Chromatin immunoprecipitation (ChIP)

Chromatin immunoprecipitation (ChIP) assays were performed essentially the same as described in [28]. Briefly, cells were fixed with 1% formaldehyde, and then whole-cell lysates were prepared. Protein lysate was subjected to ChIP with the indicated antibodies, followed by DNA purification (Invitrogen, Waltham, MA, USA). ChIP-enriched DNA was analyzed by PCR with the indicated primer sets (Table S1 in Additional file 3) purchased from NZYTech (Lisbon, Portugal). Visualization of bands was achieved using a BioRad Chemidoc XRS+ (BioRad) and quantified using the Image Lab software (BioRad).

### Quantitative real time PCR (qRT-PCR)

Total RNA was extracted by using Tri-reagent (Sigma-Aldrich). Real-time PCR was performed on a CFX-96 PCR machine (BioRad) using the SsoFastSYBR™ green master mix (BioRad) and following the manufacturer’s guidelines. The primer sequences for measuring all of our genes of interest were purchased from NZYTech and are shown in Table S1 in Additional file 3. Data was analysed as described in [29] using the 2^*-ΔΔCT*^ methodology.

## Results

### Comparative analysis of the novel PI3K inhibitor ETP-45658

We have identified pyrazolopyrimidine derivatives as biochemical inhibitors of PI3K including ETP-45658 although little was known about this compound other than it can inhibit PI3K activity [17]. Our first objective was to analyse the biological activity of ETP-45658 including the physical and chemical properties of this compound compared to the reference pan-class I PI3K and mTOR inhibitor PI-103 [9]. We characterised the structure of each compound (Figure S1A in Additional file 1) and conducted the *in silico* analysis of each compound to measure their physiochemical properties (Figure S1B in Additional file 1). We determined the molecular polar surface area (PSA) of each compound, the partition coefficient, the distribution coefficient, compound solubility and the number of hydrogen donors and acceptors. Our data also indicates that ETP-45658 is more soluble in aqueous medium at acid pH (compared to PI-103) although both compounds’ permeability is similar (as indicated by an equivalent PSA value). Crucially in terms of drug development and potential development as a novel therapeutic, ETP-45658 obeys Lipinski’s Rule of Five, suggesting that ETP-45658 could serve as therapeutic agent [30]. Having determined the key characteristics of ETP-45658, we evaluated the inhibitory activity of this compound (in comparison to PI-103) against each member of the PI3K family, including PI3K class 1 isoforms in addition to distinct p110 mutants. ETP-45658 (and PI-103) inhibited PI3K and mTOR activity although PI-103 was slightly more effective against PI3K but significantly less potent at inhibiting mTOR activity compared to ETP-45658 (Figure S1C in Additional file 1). To develop this analysis further, we expanded this screen and determined the inhibitory activities of ETP-45658 and PI-103 in a panel of 24 representative kinases (Figure S1D in Additional file 1). Each indicated protein kinase activity was assayed following 10 μM treatment with either compound. Of these 24 kinases, only mutated BRAF was significantly inhibited by either agent, with an average percent inhibition of 38% following ETP-45658 treatment (compared to 57.7% following PI-103 exposure) demonstrating that ETP-45658 is a potent, highly specific PI3K inhibitor especially when compared to the reference PI3K inhibitor PI-103.

**Figure 1:**
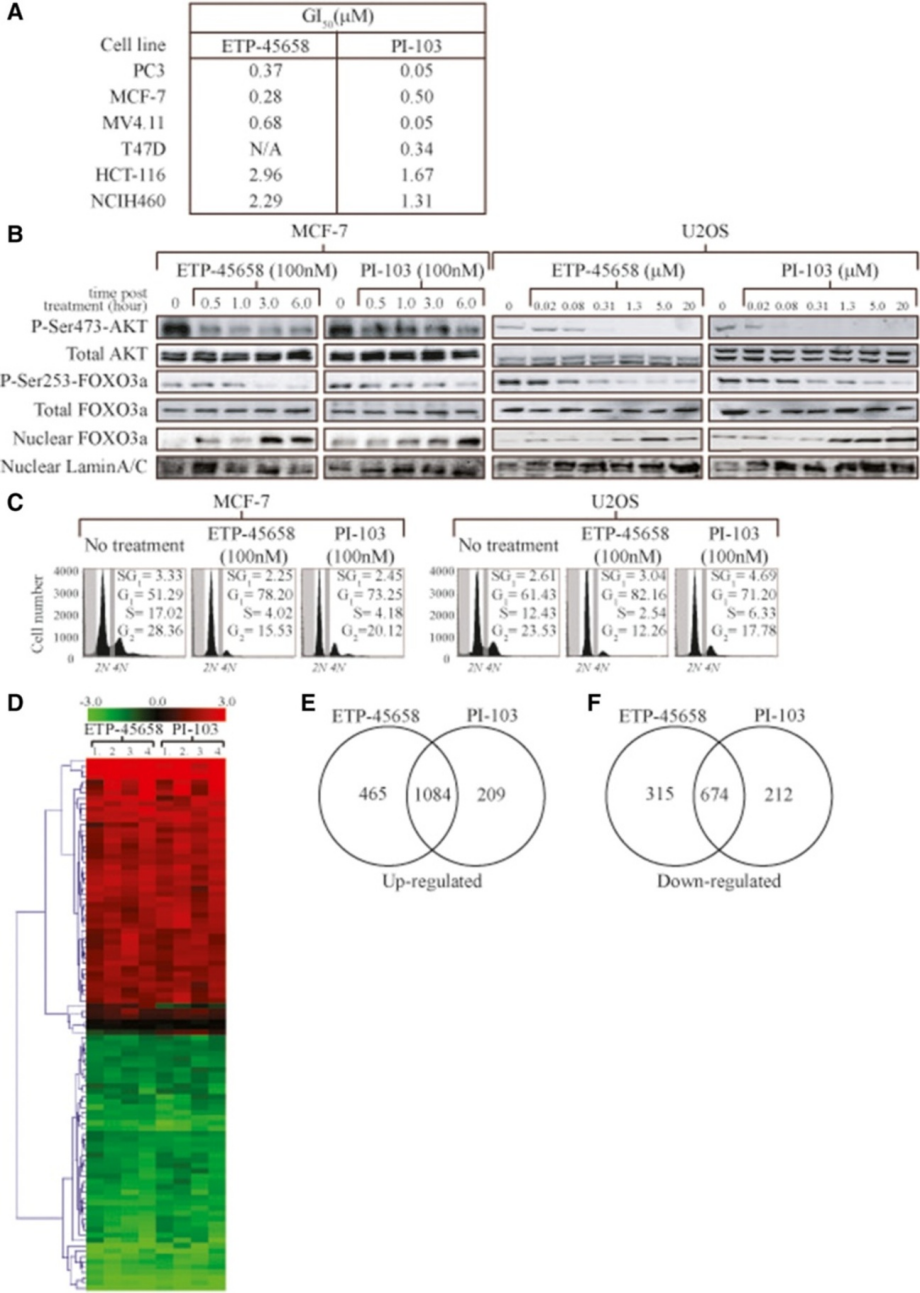
**Cellular response to ETP-45658. (A)** Proliferation assay for each indicated cell line treated with serial dilutions of ETP-45658 or reference compound PI-103. Seventy-two hours post treatment, MTT assays were conducted. **(B)** Dose- and time-dependent analysis of Ser473 phosphorylation of AKT phosphorylation by 100nM ETP-45658 or PI-103 treatment. MCF-7 or U2OS cells were treated with the indicated concentrations (μM) of ETP-45658 (or PI-103) for four hours, then total protein was extracted and immunoblotted. For nuclear FOXO analysis, nuclear protein fractions were collected four hours post ETP-45658 (or PI-103) treatment. Nuclear FOXO3a levels were measure by immunoblotting. Nuclear fractions were confirmed by probing for lamin A/C. **(C)** FACS profile of MCF-7 or U2OS following 100 nM ETP-45658 treatment (20,000 events scored, N = 5). **(D)** Heat map for the top 100 differentially expressed genes based on hierarchical clustering using Euclidean distance metric. Red and green indicate genes that induced or repressed respectively by ETP-45658 or PI-103. Black indicates no change (N = 4). **(E)** Venn diagram of upregulated genes after ETP-45658 or PI-103 treatment (100nM). **(F)** Venn diagram of downregulated genes after ETP-45658 or PI-103 exposure. Numbers indicate number of genes that are exclusive to that particular category.

We next wanted to evaluate a range of model *in vitro* cancer cell lines to determine their sensitivity to ETP-45658 treatment. We determined the EC_50_ value for the inhibition of proliferation in each of the following cell lines, PC3, MCF7, MV4.11, T47D, HCT-116 and NCIH46 cells. The inhibitory range for ETP-45658 was between 0.28 μM and 2.96 μM (and between 0.049 μM and 1.76 μM for PI-103) (Figure 1A). Of all of our tested cell lines, the MCF-7 breast cancer cell line (containing a K545E PI3K oncogenic mutation) exhibited the highest sensitivity to ETP-45658. To categorically address the ETP-45658 mode of action (as well as that of PI-103) we treated MCF-7 cells with either compound for six hours and compared the results with data obtained from experiments using the osteosarcoma cell line U2OS with a well-characterised PI3K response (Figure 1B). As early as 30 minutes post treatment with either drug, there was a significant decrease in phosphorylated serine 473 AKT and by three hours post treatment we were unable to detect this post-translational modification in treated osteosarcoma cells. Regardless of the time point investigated, there was no change in the total AKT protein level in either the breast or osteosarcoma cell lines. After observing the reduction and subsequent loss of AKT phosphorylation, we questioned if there was the concomitant downstream signalling cascade to effecter proteins following treatment with either compound. Consistent with the loss of AKT phosphorylation, we note the significant reduction of serine 253-phosphorylated FOXO3a, a key protein target that is inhibited by active AKT (Figure 1B). In contrast to the rapid loss of AKT phosphorylation, FOXO3a dephosphorylation was slower. Nevertheless after six hours post treatment there was an almost complete absence of this FOXO3a modification in either breast or osteosarcoma cancer cells. This conserved response to ETP-45658 (and PI-103) was both temporal and concentration dependent. The loss of FOXO phosphorylation indicated that AKT signalling was inhibited, however, it did not demonstrate if ETP-45658 treatment resulted in the nuclear accumulation of FOXO3a. To address this question, we collected nuclear fractions of treated cells at each time point indicated and conducted immunoblotting for FOXO3a (Figure 1B). Consistent with the inhibition of AKT and the time-dependent loss of phosphorylated FOXO3a, we detect the significant accumulation of nuclear FOXO3a. Having observed AKT inhibition, the loss of an AKT-dependent modification target and the accumulation of nuclear FOXO3a, we questioned what the cellular phenotype for the breast or osteosarcoma cells would be after treatment with ETP-45658. ETP-45658 treatment triggered a potent cell cycle arrest phenotype characterised by significant G_1_ and G_2_ peaks with little to no S-phase or sub-G_1_ cell population (Figure 1C). This cellular response was also noted following treatment with our reference drug PI-103. Having noted an arrest response, we examined the effect of ETP-45658 in the well-established breast cancer MDA-MB231 *in vitro* cell migration assay. MDA-MB231 cells were grown in a two-dimensional matrigel transwell chamber and following treatment with ETP-45658 (or PI-103), cell invasion was evaluated (Figure S1E in Additional file 1). ETP45658 (and PI-103) inhibited the capacity of MDA-MB231 cells to migrate through the matrigel without significantly affecting cell viability, confirming an anti-proliferation rather than an apoptotic response following PI3K inhibition. Our detailed analysis defining the physical, chemical and biological properties indicate the potential of ETP-45658 as a novel therapeutic agent with a highly specific mechanism of action with the strongest potency demonstrated in (although not exclusively in) breast cancer. These studies enabled us to define the experimental design for the comparative analysis for a genome-wide transcriptional study elicited after ETP-45658 treatment (Figure 1D). We observe a significant global transcriptional effect after treatment with ETP-45658 and consistent with this compound (and PI-103) inhibiting PI3K activity, we note a similar genome-wide expression profile after treatment with either drug (Additional file 4). Using a 1.5-fold expression change threshold we identified 2,538 differentially expressed genes after ETP-45658 exposure of which 1,549 genes were induced (Figure 1E) and 989 were repressed (Figure 1F). PI-103 treatment resulted in 1,293 upregulated genes and 886 downregulated genes. While there were conserved genes that were induced or repressed by each compound, a number of genes were differentially affected by each compound. However, considering the similar phenotype (Figure 1C), the conserved anti-proliferative effect and the profound effect on global gene expression after PI3K inhibition upon exposure to ETP-45658, we directed our attention towards the key genes that displayed a conserved expression after treatment between ETP-45658 and the reference compound PI-103.

### Identification of functional categories and pathways enriched in differentially regulated genes

Having noted a potent anti-proliferative effect following ETP-45658 treatment, we next questioned if there were conserved signalling cascades or a specific biological process affected after ETP-45658 treatment. We employed dual SEA and GSEA (as described in our Materials and methods section) identifying the cell cycle and cell cycle-related processes to be highly enriched in differentially regulated genes following ETP-45658 treatment (Figure S3A, 3B in Additional file 5 and Table S2 in Additional file 2) suggesting that the cell cycle is the biological process that is the most affected after exposure to ETP-45658, a result that is supported by our FACS analysis shown previously. Interestingly, chromatin modification, DNA damage response and DNA repair genes were also upregulated following exposure to either compound. Unexpectedly, we note that pro-apoptotic genes either remained unchanged or were downregulated after exposure to ETP-45658 (Figure S3A, S3B in Additional file 5 and Table S3 in Additional file 6). These results are consistent with our observation that apoptosis was not the primary response of any of our cancer cell lines including our MCF-7 or MDA-MBA231 breast cancer cells after exposure to ETP-45658.

**Figure 2:**
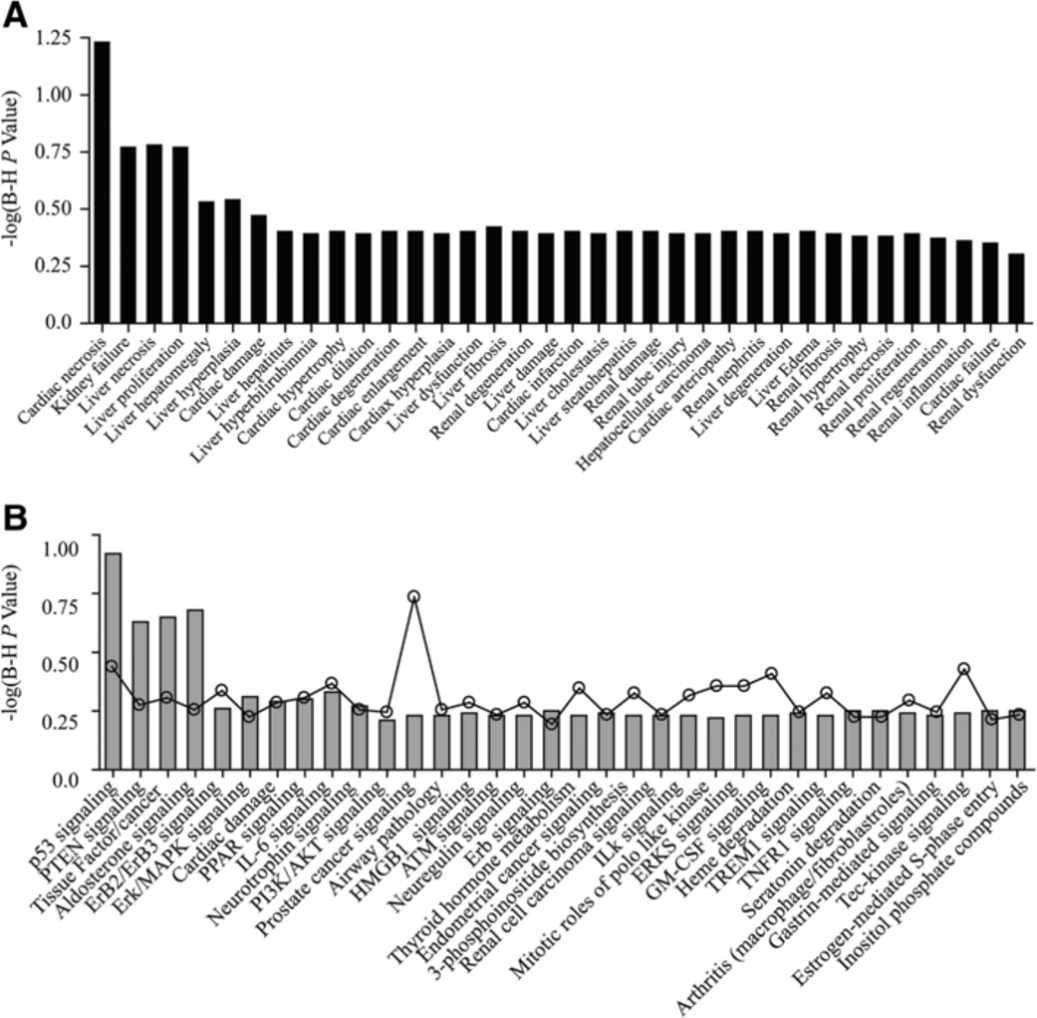
**Focused toxicity and safety assessment of ETP-45658. (A)** Analysis of BioFunctions using Ingenuity Pathway Tox Analysis comparing ETP-45658 vs DMSO (sorted by *P* value where Y axis represents -log (*P* value). **(B)** Analysis of canonical pathways using Ingenuity Pathway Tox Analysis comparing ETP-45658 vs DMSO (sorted by *P* value). Y axis represents -log (*P* value). The line graph indicates the canonical pathway ratio (number of molecules in a given pathway that meet cut-off criteria, divided by total number of molecules that make up that pathway). None of the canonical pathways have been identified as significant suggesting little or no toxicity of treatment.

While our results highlight that the primary cellular response is an anti-proliferative response (as opposed to an apoptotic response), for the development of ETP-45658 as a potential therapeutic, we questioned if ETP-45658 exposure was associated with any degree of toxicity, one of the most fundamental questions for any proposed therapeutic. We conducted a genome-wide analysis utilising an Ingenuity Pathway Analysis algorithm to address if any known toxicity pathway(s) were activated or induced after treatment with ETP-45658 (or by the reference compound PI-103). This included an extensive evaluation of the cardiac necrosis stress cascade, genes implicated in kidney failure, liver necrosis and liver hematomegaly. Furthermore, we also questioned if, on a genome-wide scale, any know canonical stress pathways were activated by ETP-45658 (or PI-103) treatment. This included the p53 pathway, PTEN aldosterone, ErbB2-ErbB3 and the ERK-MAPK signalling cascades. Neither ETP-45658 (nor PI-103) treatment significantly induced any known toxicity pathways (Figure 2A and 2B) suggesting that ETP-45658 treatment could be associated with a highly acceptable toxicity profile, particularly when compared to many chemotherapeutics in use today.

### Transcription factor enrichment analysis

**Figure 3:**
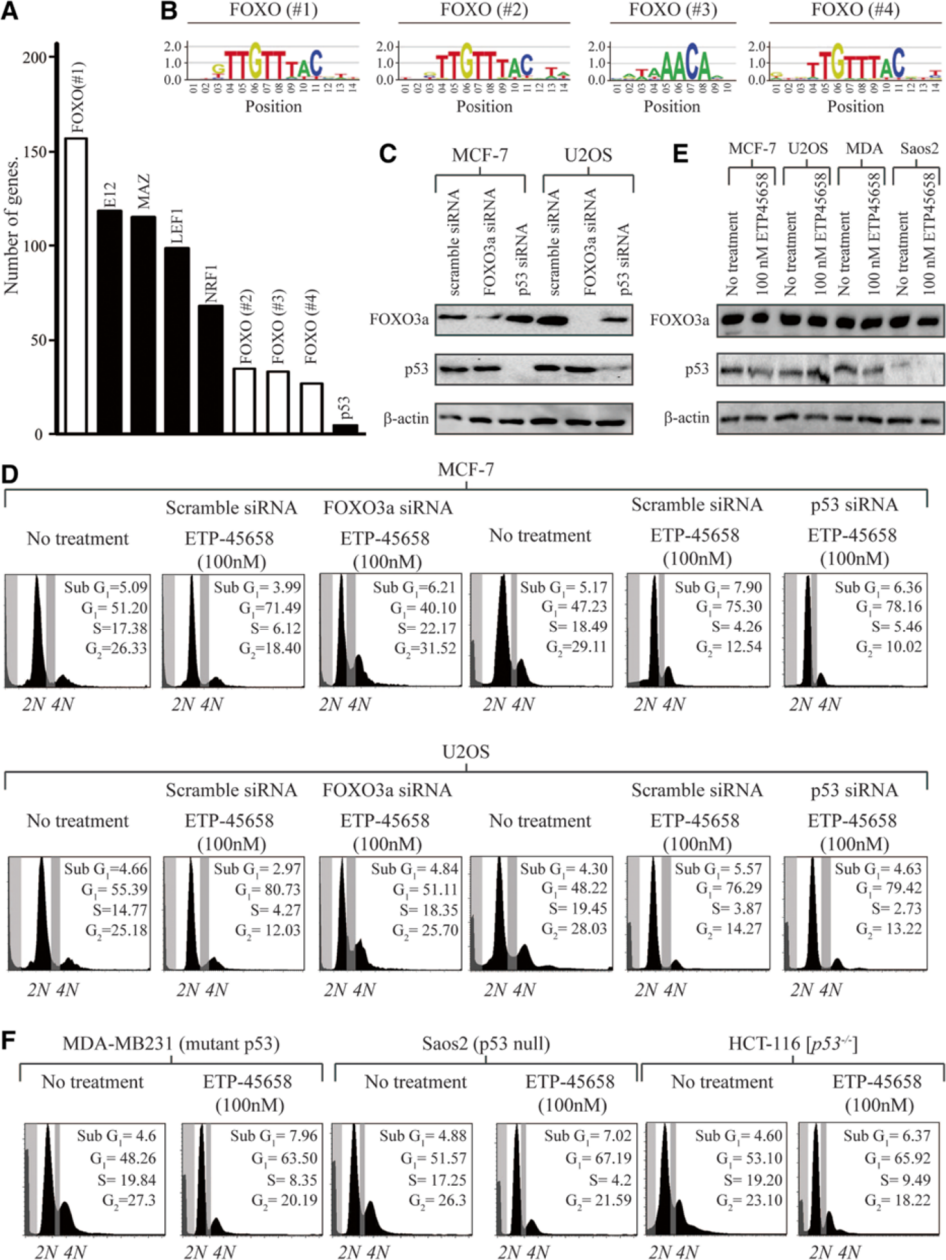
**Transcription factor analysis. (A)** Graph of transcription factors enriched in significantly upregulated genes following ETP-45658 treatment. The white bars indicate the motifs analysed in 3B). **(B)** Analysis of conserved sequence motifs for FOXO transcription factors (white bars in 3A) showing the enrichment of FOXO-binding sites within our genome-wide screen. The TRANSFAC matrix table entries for the indicated FOXO-binding sites are M00472, M00477, M00474 and M00473 respectively. **(C)** Representative immunoblots showing the effectiveness of FOXO3a or p53 knockdown in MCF-7 or U2OS cell lines following selection. **(D)** Cell cycle profile 48 hours post 100 nM ETP-45658 treatment in MCF-7- or U2OS-treated cells, N = 4. **(E)** Immunoblot showing little to no p53 accumulation following ETP-45658 exposure. **(F)** FACS analysis following 100 nM ETP-45658 treatment in MDA-MB231, Saos2 or (*p53* ^*−/−*^) HCT-116 cells N = 4.

Our data thus far indicates that ETP-45658 (as well as a PI-103) exerts its effect by inhibiting the cell cycle. However the PI3K signalling pathway can also direct a plethora of other cellular functions and responses by the regulation of multiple transcription factors that includes FOXO [16],[31], p53 [32], YAP [33], NFkB [34], CREB [35],[36], c-MYC [37],[38], and c-JUN [39]. To address which transcription factor(s) directed the cellular response to ETP-45658 treatment, we carried out an enrichment analysis for transcription factor binding sites based on our genome-wide array data sets. Our studies identified binding sites for FOXOs, E12, MAZ, LEF1 and NRF1, to be enriched and consistent with the toxicity analysis we conducted, we did not observe any binding site enrichment for p53, NFkB or c-JUN (Figure 3A). The significant accumulation of FOXO-dependent genes led us to question if there was an equivalent enrichment of FOXO-bound DNA motifs. A transcription factor enrichment analysis demonstrated that there was a highly significant increase of bound FOXO sites (Figure 3B). We questioned if the enrichment of these transcription factors was significant. We calculated *P* values for each enriched motif and noted that our *P* value range for these increased motifs were between 8.24E-14 and 5.55E-16, indicating that the increased binding shown in Figure 3A and 3B was extremely significant.

### ETP-45658 treatment triggers FOXO-dependent cell cycle arrest independent of p53

**Figure 4:**
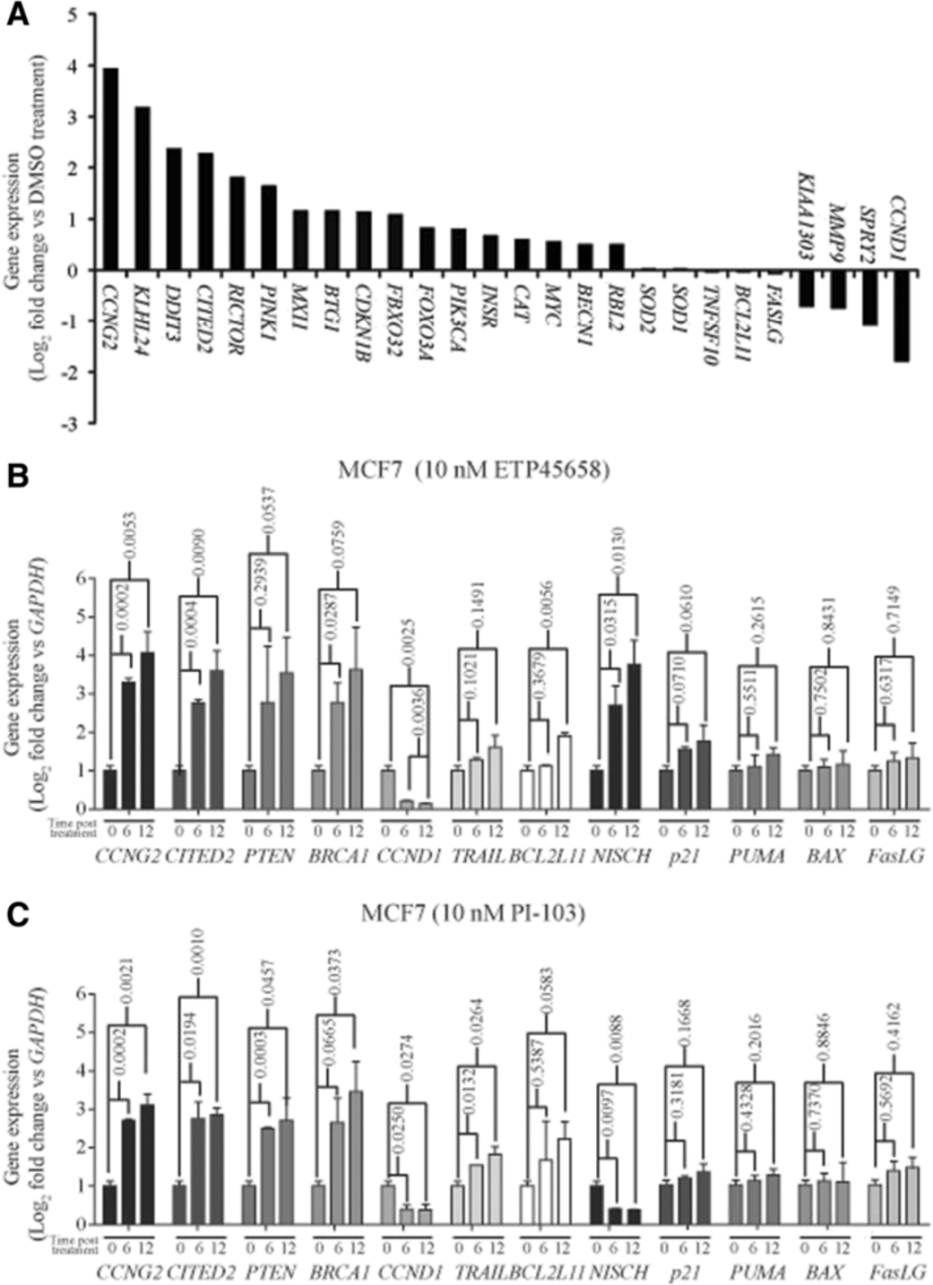
**Genes regulated by FOXO transcription factor and validation of key differentially regulated genes. (A)** Graph showing FOXO target genes that are upregulated (log2 fold change ≥ +1.0) and or downregulated (log2 fold change ≤ −1.0) following ETP-45658 (or PI-103) treatment in MCF-7 treated breast cancer cells. Gene expression in MCF-7 breast cancer cells treated at six and twelve hours with **(B)** 10 nM ETP-45658 or **(C)** 10 nM PI-103. In each graph, error bars indicate +/− standard deviation with each gene and condition evaluated in triplicate from N = 3 independent experiments. For each analysis *P* values are shown (<0.05 is considered significant).

Having noted a potent cell cycle arrest response following ETP-455658 (or PI-103) treatment, no enrichment of p53 DNA binding and a significant enrichment of FOXO transcription factors, we questioned if the cell cycle arrest response was indeed FOXO dependent and p53 independent. To address this question, we knocked FOXO3a or p53 down using small interfering RNA (siRNA) in MCF7 and U2OS cells prior to ETP-45658 treatment (Figure 3C). We note that 48 hours post exposure to ETP-45658 cells that did not express FOXO3a show a significantly higher S-phase cell population and that, in contrast to breast or osteosarcoma cells that express endogenous levels of FOXO3a, the absence of a potent cell cycle arrest phenotype (Figure 3D). In contrast to cells that lack FOXO3a, the cellular status of p53 had little to no impact regarding the response to ETP-45658. Following the knockdown of p53 and ETP-45658 treatment, we note that 48 hours post treatment that there is both a robust G_1_ and G_2_ peak in addition to a significantly reduced S-phase population (Figure 3D). There was no statistically significant difference between either scramble or p53-siRNA-treated cells following ETP-45658 treatment. As a control, we note that following 5-fluorouracil (5-FU) treatment, that there is a potent p53-dependent cell cycle arrest response (independent of FOXO3a, data not shown). Furthermore and consistent with the absence of an increase of p53-dependent genes or an enrichment of p53-bound DNA, neither ETP-45658 (nor PI-103) triggered an accumulation of total p53 (Figure 4E). To categorically confirm that the cellular response is independent of p53, we broadened our study to include the MDA-MB231 (mutant p53) breast cancer, Soas2 (p53 null) osteosarcoma and (*p53* ^*−/−*^) HCT-116 colon cancer cell lines. As we predicted, these cell lines show a potent cell cycle arrest response to ETP-45658 (Figure 3F) independent of p53. These studies (as well as our invasion studies shown in Figure S1E in Additional file 1) indicate that the anti-proliferate response to ETP-45658 (and PI-103) is FOXO dependent and independent of p53.

**Figure 5:**
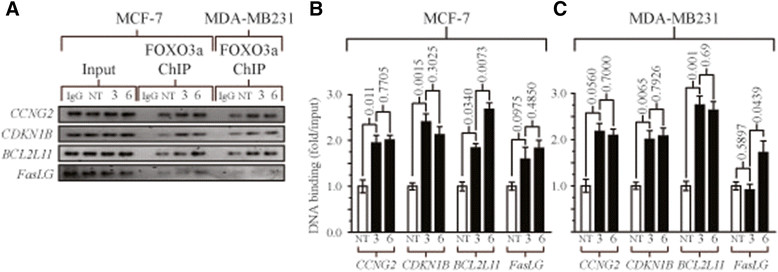
**Chromatin immunoprecipitation analysis for FOXO3a promoter recruitment. (A)** Chromatin immunoprecipitation (ChIP) assays for FOXO3a following ETP-45658 treatment at three or six hours of MCF-7 or MDA-MB231 cells for *CCG2, CDKN1B, BCL2L11*or *FASLG*. IgG indicates no specific antibody used and for simplicity, only inputs from our MCF-7 samples are shown. **(B** **and** **C)** Quantification of our ChIP gels normalised to inputs from each respective cell line. Error bars indicate standard deviation, N = 3.

After noting the significantly increased level of FOXO recruitment to its consensus sequences after treatment with ETP-45658 and a significant phenotype change following the siRNA knockdown of FOXO3a, we analysed our genome-wide array for all FOXO-dependent genes [39]-[42]. Strikingly, we note a differential induction of only a subset of FOXO-regulated genes (Figure 4A). Consistent with the enrichment of FOXO transcription factors, we observed that *CCNG2, KLHL24, DDIT3, CITED2* and *RICTOR* were significantly upregulated. In contrast *CCND1, SPRY2* and *MMP9* expression were significantly downregulated following ETP-45658 (or PI-103) treatment. Unexpectedly despite FOXO dephosphorylation, nuclear accumulation and transcription factor enrichment, the expression of FOXO-dependent target genes predominantly associated with FOXO-mediated apoptosis (including *TNFSF10, BCL211* and *FASLG*) did not significantly change after ETP-45658 (or PI-103) exposure (Figure 5A). This was also observed for the FOXO target genes *SOD1* and *SOD2* that are known to be involved in resistance to oxidative stress [43]. Consistent with this result the most upregulated gene in our entire data set was *cyclin G2 (CCNG2)*, a FOXO-regulated gene that encodes an atypical cyclin that blocks cell cycle progression [44]. In contrast, the most significantly downregulated FOXO-dependent gene was *cyclin D1 (CCND1)* a cyclin that promotes the cell cycle and cell division. Overall our data indicates that treatment of MCF-7 breast cancer cells with ETP-45658 directs the differential regulation of a specific subset of FOXO target genes, specifically targeting those associated with the cell cycle resulting in the cell cycle arrest response we observe.

### Validation of transcription profiles after ETP-45658 or PI-103 treatment of breast cancer cells

Our genome-wide array and *in vitro* studies have elucidated that the predominant pathway activated following exposure to ETP-45658 (as well as following PI-103 treatment) was FOXO-mediated cell cycle arrest. We showed the concomitant loss of FOXO phosphorylation, nuclear FOXO accumulation, the induction of FOXO-regulated genes, the absence of an arrest phenotype when FOXO3a was knocked down and have also confirmed that this cell cycle arrest response was independent of p53. To strengthen our findings further, we validated our genome-wide array results by quantitative real-time PCR (qRT-PCR). Based on our gene expression profiling data, we selected 11 differentially regulated key genes. This included *cyclin G2* (*CCNG2*)*,* the most induced gene in our array set and is FOXO regulated*, Cbp/p300-interacting transactivator* (*CITED2*)*, phosphatase and tensin homologue* (*PTEN*)*, breast cancer 1, early onset* (*BRCA1*), all three significantly transcribed after exposure to ETP-45658. We also selected *cyclin D1* (*CCND1*) that was significantly downregulated following exposure to ETP-45658. In addition to these genes, we evaluated *tumour necrosis factor (ligand) superfamily, member 10* (*TRAIL*)*, BCL2-like 11* (*BCL2L11*)*, Fas ligand* (*FASLG*) which are FOXO-dependent genes that do not show any significant transcriptional change following treatment with ETP-45658. We selected *nischarin* (*NISCH*) as this gene was significantly induced by ETP-45658 although significantly downregulated by PI-103 treatment. In our last group, we selected the *cyclin-dependent kinase inhibitor 1A* (*p21*), which is regulated by both FOXO and p53*, BCL2 binding component 3* (*PUMA*) and *BCL2-associated X* (*BAX*) that are regulated by p53. MCF-7 breast cancer cells were treated with ETP-45658 and six or twelve hours post treatment the total RNA was extracted. We evaluated the expression profile of each gene (normalised to *GAPDH* using the 2^*-ΔΔCT*^ method described in our Materials and methods section) (Figure 4B). Consistent with our array studies we note that there was a highly significant induction of *CCNG2, Cited2* and *BRCA1* with the most potent induction being observed for *CCNG2*. This response was conserved between the MCF-7 breast cancer cell line (as we would predict based on our array studies) as well as the U2OS osteosarcoma cell line (data not shown). Our qRT-PCR studies also confirmed that *CCND1* transcription was significantly reduced following ETP-45658 treatment (as well as after PI-103 exposure). As we would have predicted from our microarray studies, qRT-PCR analysis detected no significant change in *TRAIL, FASLG* or *BCL2L11* transcription six hours post ETP-45658 treatment, however, it did show a slight increase (less than two-fold) in transcription of each gene at twelve hours post treatment. Interestingly, our array screen highlighted that, in contrast to PI-103, ETP-45658 induced the significant transcription of *NISCH*. This was also independently validated in our qRT-PCR studies demonstrating a very strong correlation between our array data and our qRT-PCR experiments. Consistent with our data indicating a p53-independent response, there was no significant induction of any p53-dependent gene we evaluated and conclude that the accumulation of CDKN1A/*p21* expression we observe is likely FOXO dependent [45]-[47]. Taken together, these data indicate that ETP-45658 (and PI-103), induce a potent FOXO-dependent, p53-independent cell cycle arrest response, characterised with little to no FOXO-mediated apoptotic gene induction that was conserved in breast and osteosarcoma cell lines.

### Differential FOXO-dependent gene expression is not due to ablated promoter binding

Our results highlighted that despite inhibiting AKT, triggering FOXO nuclear accumulation and mediating a potent FOXO-dependent cell cycle arrest response, following ETP-45658 treatment that there is a clear differential expression profile of FOXO-regulated genes, with little to no pro-apoptotic FOXO-gene expression in contrast to the robust expression of FOXO-dependent cell cycle arrest genes. This led us to hypothesise that this could be the result of differential FOXO-promoter binding. To answer this question, we conducted ChIP time courses up to six hours post ETP-45658 treatment in either MCF-7 or MDA-MB231 breast cancer cells (Figure 5). For this study, we selected the *CCGN2* and *cyclin-dependent kinase inhibitor 1B* (*CDKN1B*) cell cycle arrest genes (the first being FOXO dependent and the most strongly induced gene in our study while the second encodes a FOXO-dependent cell cycle arrest protein. We also selected *BCL2L11* and *FASLG* as both are FOXO-dependent pro-apoptotic genes that show no significant transcription changes at six or twelve hours post ETP-45658 exposure. Strikingly, we note that despite the clear differential gene expression profiles noted between the four genes, there was statistically significant FOXO3a promoter recruitment for each gene examined by six hours post ETP-45658 treatment (Figure 5B and 5C). This suggests that while an absence of FOXO3a promoter binding could account for the preferential cell cycle arrest gene expression profile, that this is not the case and that there is robust pro-apoptotic promoter recruitment. This suggests that the disparity between cell cycle arrest and pro-apoptotic FOXO genes arises after FOXO binding.

## Discussion

The gene encoding p110α (*PIK3CA*) is one of the most commonly mutated kinase in the human genome [9]. Somatic missense mutations in *PIK3CA* are found in approximately 15% of all human cancers. In addition *PIK3CA* mutations are the most common genetic aberrations observed in breast cancer and occur most frequently in HER2-amplified and hormone-receptor-positive breast cancers [48]-[50]. Given the importance of PI3K anomalies in breast cancer, the pharmaceutical inhibition of PI3K has received significant attention. Here we characterise a novel therapeutic ETP-45658 and report the effect following PI3K inhibition on the global gene expression profile in the MCF-7 model breast cancer cell line that contains an oncogenic missense mutation in *PIK3CA*. The pyrazolopyrimidine derivative ETP-45658 is a potent inhibitor of PI3K that directs the nuclear accumulation of FOXO proteins. We evaluated the key properties of ETP-45658 (in comparison to the increasingly used reference PI3K inhibitor PI-103) [9] and show that ETP-45658 potently inhibited class I PI3Ks and mTOR. The observation that ETP-45658 is almost three times more potent than the current reference compound PI-103 against mTOR could be extremely important in a clinical setting where limited polypharmacology can be advantageous [51]. Furthermore, mTOR activity has been shown to be essential for PI3K-dependent oncogenesis and is involved in feedback activation of PI3K/AKT signalling via S6 kinase and insulin receptor substrate-1 [52]-[54]. As a result compounds that target both PI3K and mTOR kinase activities at the same time are thought to inhibit PI3K/AKT signalling more efficiently than selective PI3K inhibitors but could have increased toxicity. Our detailed analysis addressed the specificity of ETP-45658, analysing the effect in a broad panel of kinases that represent almost all key families within the human kinome. The compound PI-103 was precluded from clinical development due to a number of significant liabilities that included its limited aqueous solubility [55]. Crucially, we report that ETP-45658 displays a significantly higher solubility in aqueous medium at acid pH than PI-103 indicating that ETP-45658 is considerably more suitable for *in vivo* experiments (currently underway) and warrants further clinical development. We show that ETP-45658 treatment resulted in a rapid and potent reduction of AKT phosphorylation on serine residue 473 and the concomitant reduction of FOXO3a phosphorylation. Consistent with this loss of FOXO3a phosphorylation, we also observed the significant accumulation of nuclear FOXO3a and that these responses were conserved in a range of *in vitro* cancer models, including MCF-7 and MDA-MB231 breast cancer cell line models.

Our genome-wide analysis was conducted using MCF-7 breast cancer cells that have been treated with ETP-45658, PI-103 or DMSO. The MCF-7 cell line is wild-type TP53, expresses estrogen and progesterone receptors (ER and PR respectively), and carries a GATA3 frame shift mutation [56]. Microarray gene expression analysis identified more than 2,000 differentially regulated genes after we treated our cells with ETP-45658 (or PI-103) reflecting the major impact of PI3K inhibition on the transcriptional activity of genes. The number of conserved genes between these drugs was 1,760. Given the common mechanism of action between ETP-45658 and PI-103, this significant overlap was expected and confirms the consistency of our experiments. We placed these changes into a biological framework reporting here that the most significantly affected biological response by ETP-45658 (and PI-103) was the cell cycle and cell cycle-related processes. This is in agreement with our presented data here as well as previous reports that showed growth arrest occurred in the absence of apoptosis following glioma cell treatment with PI-103 or oesteosarcoma cell treatment with ETP-45658 [17],[57]. We also asked if any known toxicity or stress signalling cascades were triggered after exposure to ETP-45658 and crucially, we note that there was no significant induction of any of the pathways or cascades that we investigated. We also showed that binding sites for members of the FOXO family of transcription factors were highly enriched in our studies although it remains to be elucidated how PI3K inhibition is so efficient at mediating FOXO activation in MCF-7 cells that present a low basal level of AKT activity. Strikingly, upon ETP-45658 treatment MCF-7 (and U2OS) cells only induce a subset of these FOXO target genes. Specifically, only the cell cycle-related FOXO-dependent genes presented an altered transcriptional response, whereas stress resistance or pro-apoptotic FOXO-dependent genes were found mostly unaffected by ETP-45658 (or PI-103) treatment. While these pro-apoptotic genes were not significantly induced, there was robust FOXO protein recruitment for each gene that we examined by ChIP, indicating that this transcriptional response is regulated post-FOXO DNA recruitment. Hence the interaction with cell or context-specific co-factors, rather than specific recruitment of FOXO3a to promoters of cell cycle genes may explain our observations. This is in agreement with previous studies in which several co-factors were shown to dictate the transcriptional response to FOXO activation [58]. Importantly, in colon cancer, high amount of nuclear beta-catenin interacting with FOXO3a has been reported to co-regulate metastasis-relevant genes upon PI3K/AKT pathway inhibition [59]. Accordingly, PI3K inhibition in breast tumours with high level of nuclear beta-catenin might promote metastasis. In a recent study, however, beta-catenin was shown to localise to the plasma membrane and cytoplasm in MCF7 cells [60]. The identification of specific cofactors that regulate the FOXO-mediated expression of cell cycle genes in breast cancer cells remains pertinent for a thorough understanding of FOXO biology.

An important caveat of our studies presented here is that these experiments only present a global snapshot regarding PI3K-regulated gene transcription six hours after each treatment and does not distinguish between a primary and secondary transcriptional response. A recent study by Eijkelenboom *et al*. suggests that a variation in overall chromatin architecture between different systems is responsible for the context-dependent nature of FOXO activation [40]. The analysis of FOXO-regulated genes in our data set revealed the cell cycle as the most significantly affected biological process by ETP-45658 treatment, strongly supporting the hypothesis that after the treatment with ETP-45658 (or PI-103) cell cycle arrest is likely to be mediated by the action of FOXO proteins. Indeed, this is corroborated by our FACS and proteomic studies that we present here. In addition, *cyclin G2* and *cyclin D1* were among the most upregulated and the most downregulated genes respectively. *Cyclin G2* is suppressed by PI3K/AKT signalling in proliferating cells [44] while *cyclin D1* has been identified as a target gene downregulated by FOXO and involved in the FOXO-mediated inhibition of cell cycle [61]. Importantly, we confirmed our data for many genes of interest by qRT-PCR indicating the high accuracy of our expression profiling studies. Strikingly, with both modalities, *NISCH* was found to be significantly upregulated by ETP-45658 treatment but downregulated after PI-103 treatment. *NISCH* has been established recently as a tumour suppressor in breast cancer and has significantly higher expression in normal breast tissue samples compared to tumour samples [62]. The therapeutic relevance of this result is currently under investigation within our laboratory.

As the exclusion of transcriptionally active FOXO proteins from the nucleus is a key feature of cells that have been transformed by oncogenic PI3K or AKT the restoration of FOXO activity has been suggested as a very attractive strategy to treat cancer [31],[63]. However, as FOXO factors also mediate cellular stress resistance, they might also increase resistance to chemotherapeutics. Therefore, it is crucial to characterise specific FOXO-dependent transcriptional programmes in various cell systems to specifically enable the reactivation of FOXO tumour suppressor functions.

## Conclusions

In summary, we show that inhibition of PI3K using different small molecule inhibitors potently suppresses the growth of breast cancer cells via inhibiting cell cycle and that this response is FOXO protein dependent and p53 independent. Using genome-wide microarray analysis, we found that FOXO factors are the major downstream transcriptional effectors of PI3K/AKT signalling. However, only a subset of all FOXO-dependent genes was induced upon PI3K inhibition. Intriguingly, this was not due to differential FOXO promoter recruitment, suggesting that the disparity between cell cycle arrest and pro-apoptotic FOXO target genes arises after FOXO binding.

## Additional files

## Electronic supplementary material

Additional file 1: Figure S1.: Comparative analysis of ETP-45658 and PI-103. **(A)** Chemical structure of ETP-45658 and PI-103. **(B)** Physiochemical properties of ETP-45658 and PI-103, including molecular polar surface area (PSA), partition coefficient (LogP), ACD clogD and solubility at two different pH values including Lipinsky’s Rule of Five (RoF) analysis. **(C)** Inhibitory activity against PI3K and mTOR. The kinase activity of PI3K was measured by using the commercial PI3-kinase HTRF™ assay mTOR activity by LanthaScreen™. **(D)** ETP-45658 and PI-103 screen against a panel of 24 kinases. The value shown indicates kinase inhibition (percentage +/− standard deviation) at 10 μM. **(E)** Migration of MDA-MB231 cells in a two compartment Boyden chamber for 72 hours in the presence of DMSO, ETP-45658 or PI-103 (N = 3). (JPEG 662 KB)

Additional file 2: Table S2.: Ranking analysis of all cell cycle genes. This table indicates 1,256 cell cycle-related genes following our clustering analysis. This table includes gene symbols and their corresponding Entrez gene identities in addition to their averaged log_2_ fold change and adjusted *P* values after analysing gene array data ((ETP-45658 vs DMSO) and (PI-103 vs DMSO); across all four replicates) using LIMMA. (XLSX 123 KB)

Additional file 3: Table S1.: Primer sequences for quantitative real-time PCR, siRNA targeting and ChIP assays. This table indicates the complete 5′-3′ sequences for each gene of interest shown. All sequences were obtained from NZYTech (Lisbon, Portugal). (XLSX 12 KB)

Additional file 4: Figure S2.: Gene expression changes following ETP-45658 or PI-103 treatment of MCF-7 cells. **(A)** Volcano plots showing statistical significance (−log10 *P* value) plotted against log2 fold change for either ETP-45658 vs DMSO or PI-103 vs DMSO. Each indicates significantly overexpressed genes (log2 fold change ≥+1.0, adjusted *P* value ≤0.05) in red and downregulated genes (log2 fold change ≤−1.0, adjusted *P* value ≤0.05) in green. (JPEG 788 KB)

Additional file 5: Figure S3.: Distribution of differentially regulated genes that are common to ETP-45658 and PI-103 treatments across biological processes and pathways. **(A)** Biological processes that are significantly represented in upregulated genes. **(B)** Biological processes that are enriched in significantly downregulated genes. Number refers to different regulated genes under each biological process (BP) category. Note that the BP categories are not exclusive, that is, a gene can be assigned to several BP categories. The white bars in A and B indicate the total number of genes within each section (for example the cell cycle); however, we have also shown specific facets of these processes, for example, the number of genes involved exclusively in cell cycle arrest). **(C)** Biological pathways that are enriched in significantly upregulated genes. **(D)** Biological pathways that are represented in significantly downregulated genes. Number indicates number of differentially regulated genes assigned to each pathway. (JPEG 1 MB)

Additional file 6: Table S3.: Ranking analysis of key apoptosis genes. This table shows 1,452 apoptosis-related genes used for our clustering analysis. The table includes gene symbols, their corresponding Entrez gene identities, each averaged log_2_ fold change and their adjusted *P* values following the analysis of our gene array studies ((ETP-45658 vs DMSO) and (PI-103 vs DMSO); across four replicates) using LIMMA. (XLSX 137 KB)

Below are the links to the authors’ original submitted files for images.

Authors’ original file for figure 1

Authors’ original file for figure 2

Authors’ original file for figure 3

Authors’ original file for figure 4

Authors’ original file for figure 5

## References

[1] Ferlay J, Shin HR, Bray F, Forman D, Mathers C, Parkin DM (2010). Estimates of worldwide burden of cancer in 2008: GLOBOCAN 2008. Int J Cancer.

[2] Stemke-Hale K, Gonzalez-Angulo AM, Lluch A, Neve RM, Kuo WL, Davies M, Carey M, Hu Z, Guan Y, Sahin A, Symmans WF, Pusztai L, Nolden LK, Horlings H, Berns K, Hung MC, van de Vijver MJ, Valero V, Gray JW, Bernards R, Mills GB, Hennessy BT (2008). An integrative genomic and proteomic analysis of PIK3CA, PTEN, and AKT mutations in breast cancer. Cancer Res.

[3] Carnero A, Blanco-Aparicio C, Renner O, Link W, Leal JF (2008). The PTEN/PI3K/AKT signalling pathway in cancer, therapeutic implications. Curr Cancer Drug Targets.

[4] Vanhaesebroeck B, Leevers SJ, Ahmadi K, Timms J, Katso R, Driscoll PC, Woscholski R, Parker PJ, Waterfield MD (2001). Synthesis and function of 3-phosphorylated inositol lipids. Annu Rev Biochem.

[5] Cantley LC (2002). The phosphoinositide 3-kinase pathway. Science.

[6] Vivanco I, Sawyers CL (2002). The phosphatidylinositol 3-Kinase AKT pathway in human cancer. Nat Rev Cancer.

[7] Zhao L, Vogt PK (2008). Class I PI3K in oncogenic cellular transformation. Oncogene.

[8] Myers MP, Pass I, Batty IH, Van der Kaay J, Stolarov JP, Hemmings BA, Wigler MH, Downes CP, Tonks NK (1998). The lipid phosphatase activity of PTEN is critical for its tumor supressor function. Proc Natl Acad Sci U S A.

[9] Workman P, Clarke PA, Raynaud FI, van Montfort RL (2010). Drugging the PI3 kinome: from chemical tools to drugs in the clinic. Cancer Res.

[10] Hollstein M, Sidransky D, Vogelstein B, Harris CC (1991). p53 mutations in human cancers. Science.

[11] Hennessy BT, Smith DL, Ram PT, Lu Y, Mills GB (2005). Exploiting the PI3K/AKT pathway for cancer drug discovery. Nat Rev Drug Discov.

[12] Courtney KD, Corcoran RB, Engelman JA (2010). The PI3K pathway as drug target in human cancer. J Clin Oncol.

[13] Bader AG, Kang S, Zhao L, Vogt PK (2005). Oncogenic PI3K deregulates transcription and translation. Nat Rev Cancer.

[14] Furuyama T, Nakazawa T, Nakano I, Mori N (2000). Identification of the differential distribution patterns of mRNAs and consensus binding sequences for mouse DAF-16 homologues. Biochem J.

[15] Xuan Z, Zhang MQ (2005). From worm to human: bioinformatics approaches to identify FOXO target genes. Mech Ageing Dev.

[16] Zanella F, Link W, Carnero A (2010). Understanding FOXO, new views on old transcription factors. Curr Cancer Drug Targets.

[17] Link W, Oyarzabal J, Serelde BG, Albarran MI, Rabal O, Cebria A, Alfonso P, Fominaya J, Renner O, Peregrina S, Soilán D, Ceballos PA, Hernández AI, Lorenzo M, Pevarello P, Granda TG, Kurz G, Carnero A, Bischoff JR (2009). Chemical interrogation of FOXO3a nuclear translocation identifies potent and selective inhibitors of phosphoinositide 3-kinases. J Biol Chem.

[18] ProQinase GmbH, Freiburg, Germany [http://www.proquinase.com]

[19] The R Project for Statistical Computing [http://www.r-project.org/]

[20] Bolstad BM, Irizarry RA, Astrand M, Speed TP (2003). A comparison of normalization methods for high density oligonucleotide array data based on variance and bias. Bioinformatics.

[21] Smyth GK: Linear models and empirical bayes methods for assessing differential expression in microarray experiments. *Stat Appl Genet Mol Biol* 2004, 3:Article3., 10.2202/1544-6115.1027 16646809

[22] Klipper-Aurbach Y, Wasserman M, Braunspiegel-Weintrob N, Borstein D, Peleg S, Assa S, Karp M, Benjamini Y, Hochberg Y, Laron Z (1995). Mathematical formulae for the prediction of the residual beta cell function during the first two years of disease in children and adolescents with insulin-dependent diabetes mellitus. Med Hypotheses.

[23] Kalathur RK, Hernandez-Prieto MA, Futschik ME (2012). Huntington’s disease and its therapeutic target genes: a global functional profile based on the HD Research Crossroads database. BMC Neurol.

[24] Subramanian A, Tamayo P, Mootha VK, Mukherjee S, Ebert BL, Gillette MA, Paulovich A, Pomeroy SL, Golub TR, Lander ES, Mesirov JP (2005). Gene set enrichment analysis: a knowledge-based approach for interpreting genome-wide expression profiles. Proc Natl Acad Sci U S A.

[25] Carbon S, Ireland A, Mungall CJ, Shu S, Marshall B, Lewis S (2009). AmiGO: online access to ontology and annotation data. Bioinformatics.

[26] Kanehisa M, Goto S, Sato Y, Kawashima M, Furumichi M, Tanabe M (2014). Data, information, knowledge and principle: back to metabolism in KEGG. Nucleic Acids Res.

[27] Liberzon A, Subramanian A, Pinchback R, Thorvaldsdottir H, Tamayo P, Mesirov JP (2011). Molecular signatures database (MSigDB) 3.0. Bioinformatics.

[28] Hill R, Madureira PA, Waisman DM, Lee PW (2011). DNA-PKCS binding to p53 on the p21WAF1/CIP1 promoter blocks transcription resulting in cell death. Oncotarget.

[29] Schmittgen TD, Livak KJ (2008). Analyzing real-time PCR data by the comparative C(T) method. Nat Protoc.

[30] Lipinski CA, Lombardo F, Dominy BW, Feeney PJ (2001). Experimental and computational approaches to estimate solubility and permeability in drug discovery and development settings. Adv Drug Deliv Rev.

[31] Zanella F, Dos Santos NR, Link W (2013). Moving to the core: spatiotemporal analysis of Forkhead box O (FOXO) and nuclear factor-kappaB (NF-kappaB) nuclear translocation. Traffic.

[32] Mayo LD, Donner DB (2001). A phosphatidylinositol 3-kinase/Akt pathway promotes translocation of Mdm2 from the cytoplasm to the nucleus. Proc Natl Acad Sci U S A.

[33] Basu S, Totty NF, Irwin MS, Sudol M, Downward J (2003). Akt phosphorylates the Yes-associated protein, YAP, to induce interaction with 14-3-3 and attenuation of p73-mediated apoptosis. Mol Cell.

[34] Romashkova JA, Makarov SS (1999). NF-kappaB is a target of AKT in anti-apoptotic PDGF signalling. Nature.

[35] Grimes CA, Jope RS (2001). CREB DNA binding activity is inhibited by glycogen synthase kinase-3 beta and facilitated by lithium. J Neurochem.

[36] Du K, Montminy M (1998). CREB is a regulatory target for the protein kinase Akt/PKB. J Biol Chem.

[37] Pulverer BJ, Fisher C, Vousden K, Littlewood T, Evan G, Woodgett JR (1994). Site-specific modulation of c-Myc cotransformation by residues phosphorylated in vivo. Oncogene.

[38] Lutterbach B, Hann SR (1994). Hierarchical phosphorylation at N-terminal transformation-sensitive sites in c-Myc protein is regulated by mitogens and in mitosis. Mol Cell Biol.

[39] Boyle WJ, Smeal T, Defize LH, Angel P, Woodgett JR, Karin M, Hunter T (1991). Activation of protein kinase C decreases phosphorylation of c-Jun at sites that negatively regulate its DNA-binding activity. Cell.

[40] Eijkelenboom A, Mokry M, De WE, Smits LM, Polderman PE, Van Triest MH, Van BR, Schulze A, De LW, Cuppen E, Burgering BM (2013). Genome-wide analysis of FOXO3 mediated transcription regulation through RNA polymerase II profiling. Mol Syst Biol.

[41] Greer EL, Brunet A (2005). FOXO transcription factors at the interface between longevity and tumor suppression. Oncogene.

[42] Terragni J, Graham JR, Adams KW, Schaffer ME, Tullai JW, Cooper GM (2008). Phosphatidylinositol 3-kinase signaling in proliferating cells maintains an anti-apoptotic transcriptional program mediated by inhibition of FOXO and non-canonical activation of NFkappaB transcription factors. BMC Cell Biol.

[43] Geng T, Li P, Yin X, Yan Z (2011). PGC-1alpha promotes nitric oxide antioxidant defenses and inhibits FOXO signaling against cardiac cachexia in mice. Am J Pathol.

[44] Martinez-Gac L, Marques M, Garcia Z, Campanero MR, Carrera AC (2004). Control of cyclin G2 mRNA expression by forkhead transcription factors: novel mechanism for cell cycle control by phosphoinositide 3-kinase and forkhead. Mol Cell Biol.

[45] Arden KC (2004). FoxO: linking new signaling pathways. Mol Cell.

[46] Accili D, Arden KC (2004). FoxOs at the crossroads of cellular metabolism, differentiation, and transformation. Cell.

[47] Tinkum KL, White LS, Marpegan L, Herzog E, Piwnica-Worms D, Piwnica-Worms H (2013). Forkhead box O1 (FOXO1) protein, but not p53, contributes to robust induction of p21 expression in fasted mice. J Biol Chem.

[48] Samuels Y, Wang Z, Bardelli A, Silliman N, Ptak J, Szabo S, Yan H, Gazdar A, Powell SM, Riggins GJ, Willson JK, Markowitz S, Kinzler KW, Vogelstein B, Velculescu VE (2004). High frequency of mutations of the PIK3CA gene in human cancers. Science.

[49] Campbell IG, Russell SE, Choong DY, Montgomery KG, Ciavarella ML, Hooi CS, Cristiano BE, Pearson RB, Phillips WA (2004). Mutation of the PIK3CA gene in ovarian and breast cancer. Cancer Res.

[50] Bachman KE, Argani P, Samuels Y, Silliman N, Ptak J, Szabo S, Konishi H, Karakas B, Blair BG, Lin C, Peters BA, Velculescu VE, Park BH (2004). The PIK3CA gene is mutated with high frequency in human breast cancers. Cancer Biol Ther.

[51] Collins I, Workman P (2006). New approaches to molecular cancer therapeutics. Nat Chem Biol.

[52] O’Reilly KE, Rojo F, She QB, Solit D, Mills GB, Smith D, Lane H, Hofmann F, Hicklin DJ, Ludwig DL, Baselga J, Rosen N (2006). mTOR inhibition induces upstream receptor tyrosine kinase signaling and activates Akt. Cancer Res.

[53] Harrington LS, Findlay GM, Gray A, Tolkacheva T, Wigfield S, Rebholz H, Barnett J, Leslie NR, Cheng S, Shepherd PR, Gout I, Downes CP, Lamb RF (2004). The TSC1-2 tumor suppressor controls insulin-PI3K signaling via regulation of IRS proteins. J Cell Biol.

[54] Carracedo A, Pandolfi PP (2008). The PTEN-PI3K pathway: of feedbacks and cross-talks. Oncogene.

[55] Raynaud FI, Eccles S, Clarke PA, Hayes A, Nutley B, Alix S, Henley A, Di-Stefano F, Ahmad Z, Guillard S, Guillard S, Bjerke LM, Kelland L, Valenti M, Patterson L, Gowan S, De Haven BA, Hayakawa M, Kaizawa H, Koizumi T, Ohishi T, Patel S, Saghir N, Parker P, Waterfield M, Workman P (2007). Pharmacologic characterization of a potent inhibitor of class I phosphatidylinositide 3-kinases. Cancer Res.

[56] Usary J, Llaca V, Karaca G, Presswala S, Karaca M, He X, Langerod A, Karesen R, Oh DS, Dressler LG, Lonning PE, Strausberg RL, Chanock S, Borresen-Dale AL, Perou CM (2004). Mutation of GATA3 in human breast tumors. Oncogene.

[57] Guillard S, Clarke PA, Te PR, Mohri Z, Bjerke L, Valenti M, Raynaud F, Eccles SA, Workman P (2009). Molecular pharmacology of phosphatidylinositol 3-kinase inhibition in human glioma. Cell Cycle.

[58] van der Vos KE, Coffer PJ (2011). The extending network of FOXO transcriptional target genes. Antioxid Redox Signal.

[59] Tenbaum SP, Ordonez-Moran P, Puig I, Chicote I, Arques O, Landolfi S, Fernandez Y, Herance JR, Gispert JD, Mendizabal L, Aguilar S, Cajal S, Schwartz S, Vivancos A, Espin E, Rojas S, Baselga J, Tabernero J, Munoz A, Palmer HG (2012). Beta-catenin confers resistance to PI3K and AKT inhibitors and subverts FOXO3a to promote metastasis in colon cancer. Nat Med.

[60] Rosso M, Lapyckyj L, Amiano N, Besso MJ, Sanchez M, Chuluyan E, Vazquez-Levin MH (2014). Secretory leukocyte protease inhibitor (SLPI) expression downregulates E-cadherin, induces beta-catenin re-localisation and triggers apoptosis-related events in breast cancer cells. Biol Cell.

[61] Schmidt M, Fernandez de Mattos S, van der Horst A, Klompmaker R, Kops GJ, Lam EW, Burgering BM, Medema RH (2002). Cell cycle inhibition by FoxO forkhead transcription factors involves downregulation of cyclin D. Mol Cell Biol.

[62] Baranwal S, Wang Y, Rathinam R, Lee J, Jin L, McGoey R, Pylayeva Y, Giancotti F, Blobe GC, Alahari SK (2011). Molecular characterization of the tumor-suppressive function of nischarin in breast cancer. J Natl Cancer Inst.

[63] Hill R, Cautain B, De PN, Link W (2014). Targeting nucleocytoplasmic transport in cancer therapy. Oncotarget.

