# Supplementary figures and images for: A novel phosphatidylinositol 3-kinase (PI3K) inhibitor directs a potent FOXO-dependent, p53-independent cell cycle arrest phenotype characterized by the differential induction of a subset of FOXO-regulated genes
Source: Breast Cancer Res. 2014 Dec 9;16:482. doi: 10.1186/s13058-014-0482-y (PMC4303209; doi:10.1186/s13058-014-0482-y)

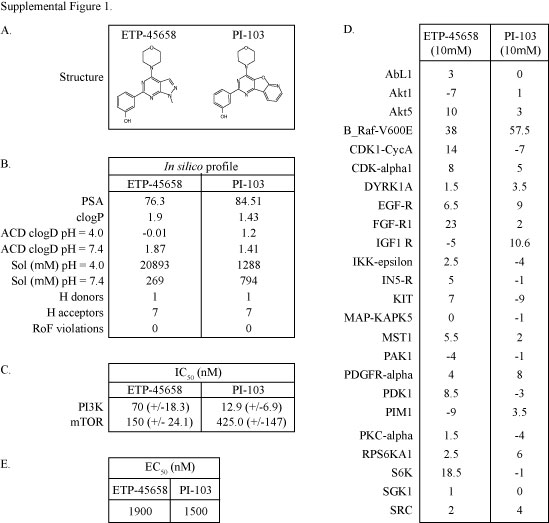

Supplement: Supplementary file 1 — Additional file 1: Figure S1.: Comparative analysis of ETP-45658 and PI-103. (A) Chemical structure of ETP-45658 and PI-103. (B) Physiochemical properties of ETP-45658 and PI-103, including molecular polar surface area (PSA), partition coefficient (LogP), ACD clogD and solubility at two different pH values including Lipinsky’s Rule of Five (RoF) analysis. (C) Inhibitory activity against PI3K and mTOR. The kinase activity of PI3K was measured by using the commercial PI3-kinase HTRF™ assay mTOR activity by LanthaScreen™. (D) ETP-45658 and PI-103 screen against a panel of 24 kinases. The value shown indicates kinase inhibition (percentage +/− standard deviation) at 10 μM. (E) Migration of MDA-MB231 cells in a two compartment Boyden chamber for 72 hours in the presence of DMSO, ETP-45658 or PI-103 (N = 3). (JPEG 662 KB) [file 13058_2014_482_MOESM1_ESM.jpeg]

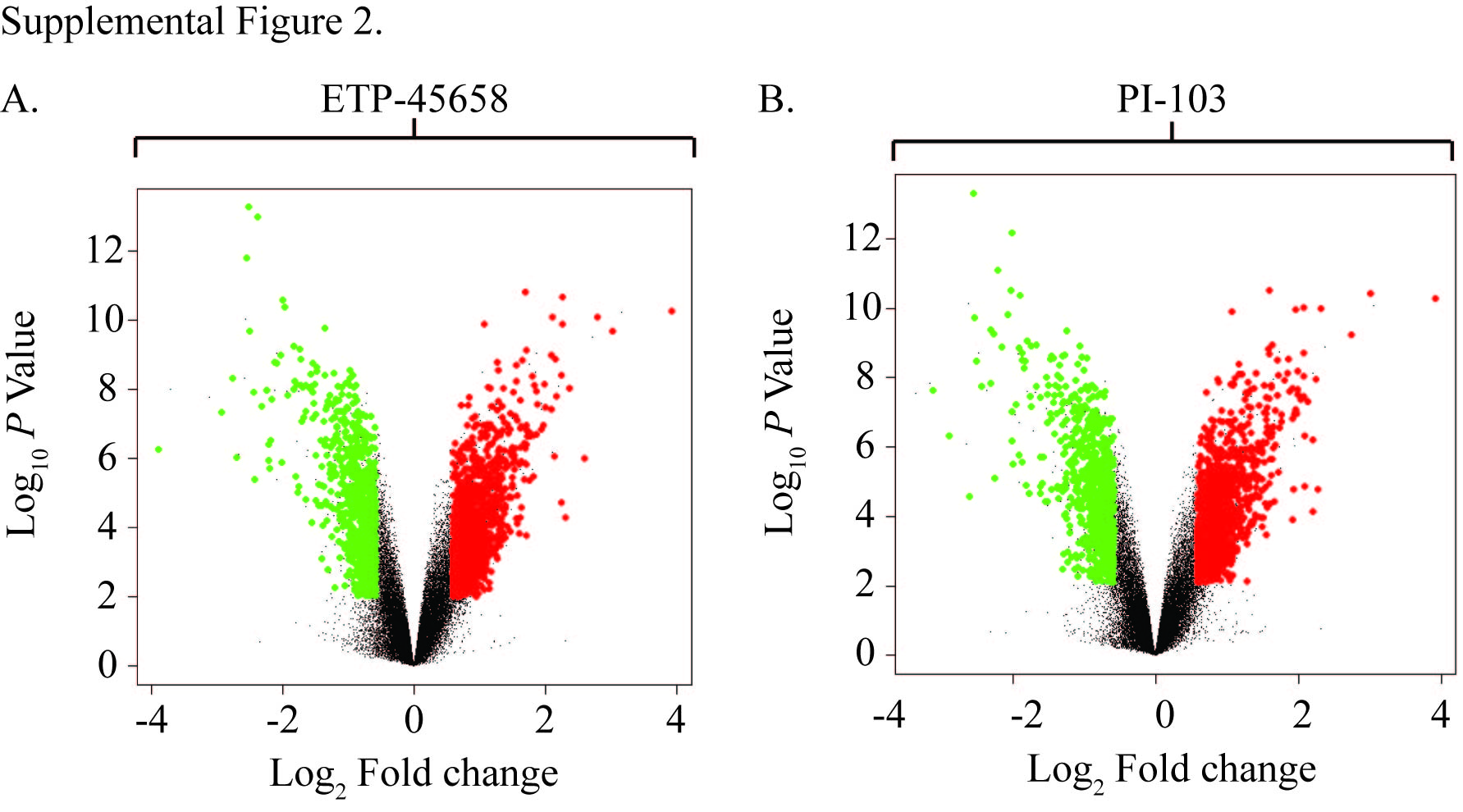

Supplement: Supplementary file 4 — Additional file 4: Figure S2.: Gene expression changes following ETP-45658 or PI-103 treatment of MCF-7 cells. (A) Volcano plots showing statistical significance (−log10 P value) plotted against log2 fold change for either ETP-45658 vs DMSO or PI-103 vs DMSO. Each indicates significantly overexpressed genes (log2 fold change ≥+1.0, adjusted P value ≤0.05) in red and downregulated genes (log2 fold change ≤−1.0, adjusted P value ≤0.05) in green. (JPEG 788 KB) [file 13058_2014_482_MOESM4_ESM.jpeg]

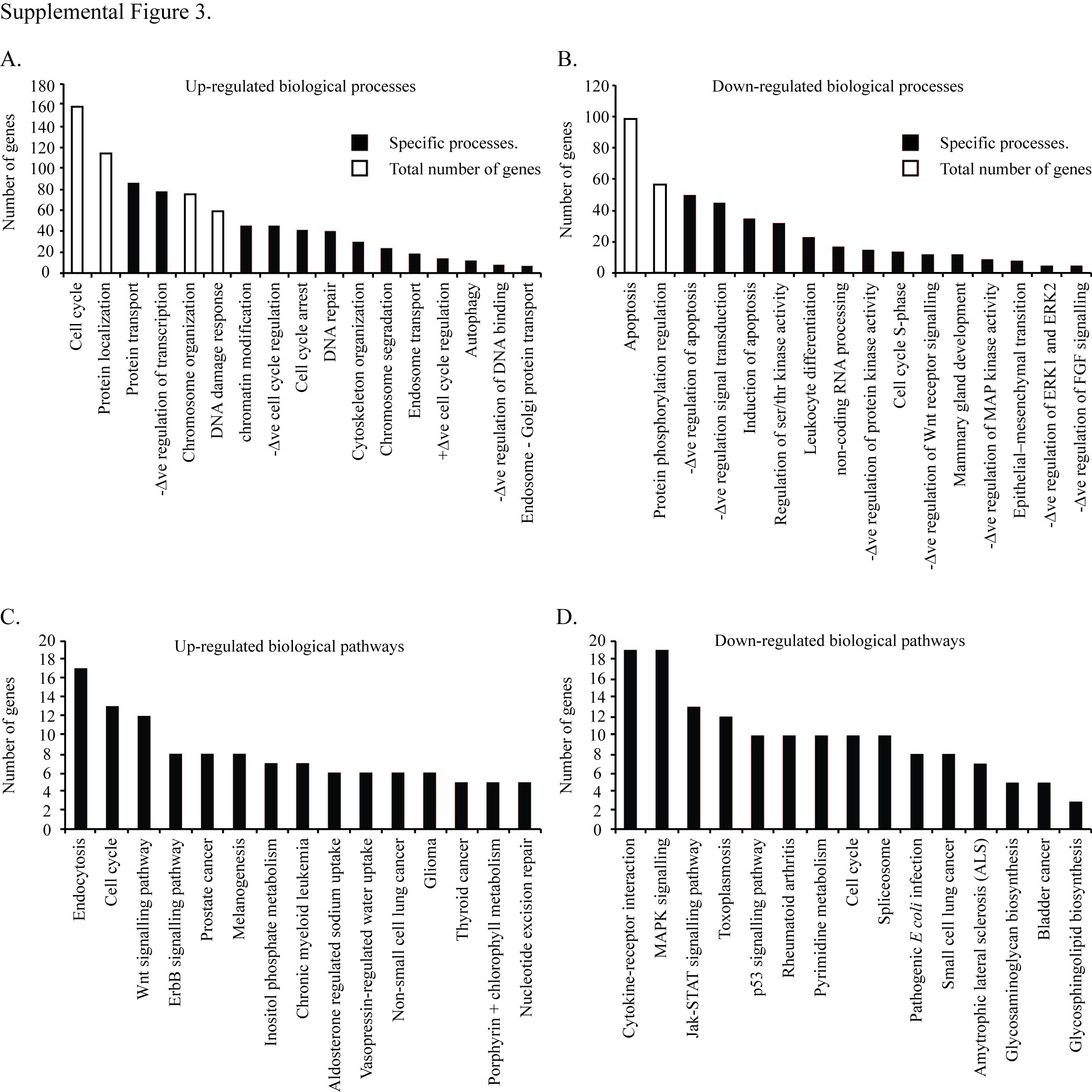

Supplement: Supplementary file 5 — Additional file 5: Figure S3.: Distribution of differentially regulated genes that are common to ETP-45658 and PI-103 treatments across biological processes and pathways. (A) Biological processes that are significantly represented in upregulated genes. (B) Biological processes that are enriched in significantly downregulated genes. Number refers to different regulated genes under each biological process (BP) category. Note that the BP categories are not exclusive, that is, a gene can be assigned to several BP categories. The white bars in A and B indicate the total number of genes within each section (for example the cell cycle); however, we have also shown specific facets of these processes, for example, the number of genes involved exclusively in cell cycle arrest). (C) Biological pathways that are enriched in significantly upregulated genes. (D) Biological pathways that are represented in significantly downregulated genes. Number indicates number of differentially regulated genes assigned to each pathway. (JPEG 1 MB) [file 13058_2014_482_MOESM5_ESM.jpeg]

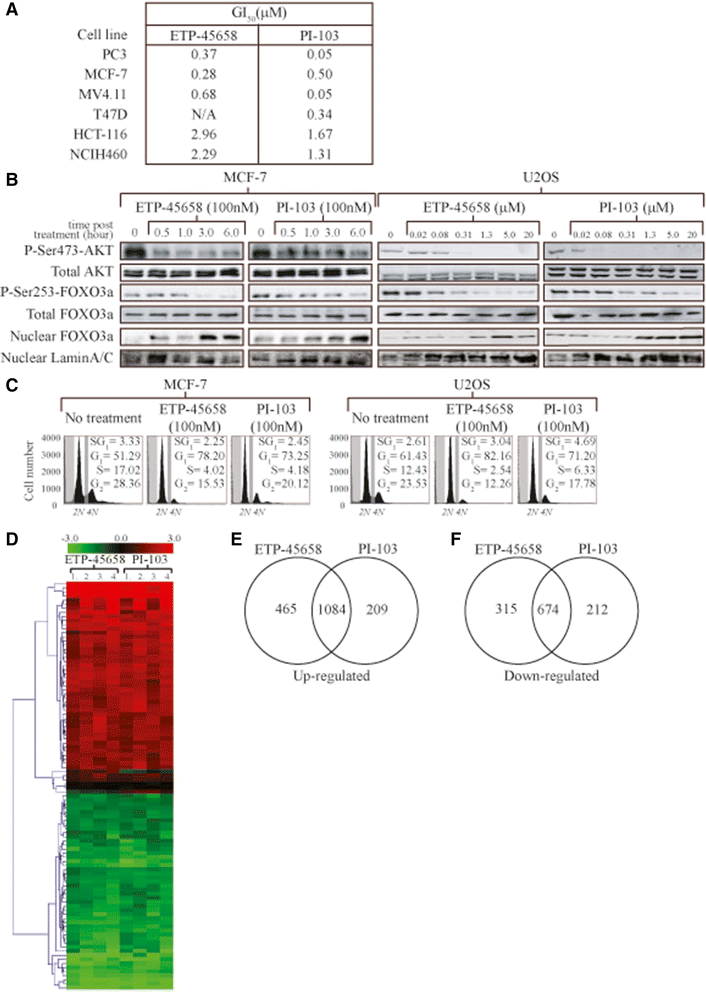

Supplement: Supplementary file 7 — Authors’ original file for figure 1 [file 13058_2014_482_MOESM7_ESM.gif]

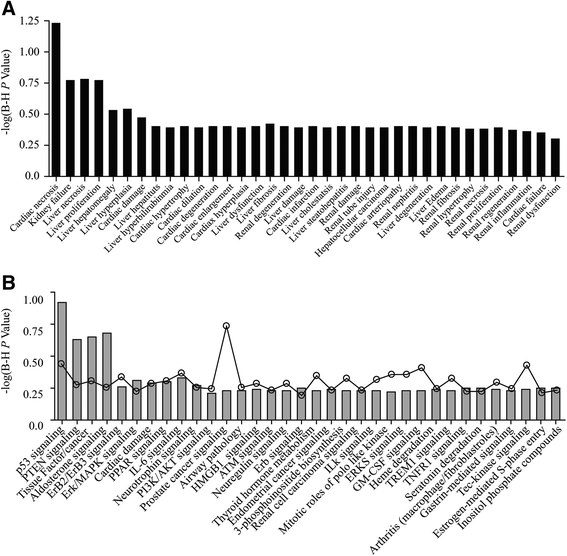

Supplement: Supplementary file 8 — Authors’ original file for figure 2 [file 13058_2014_482_MOESM8_ESM.gif]

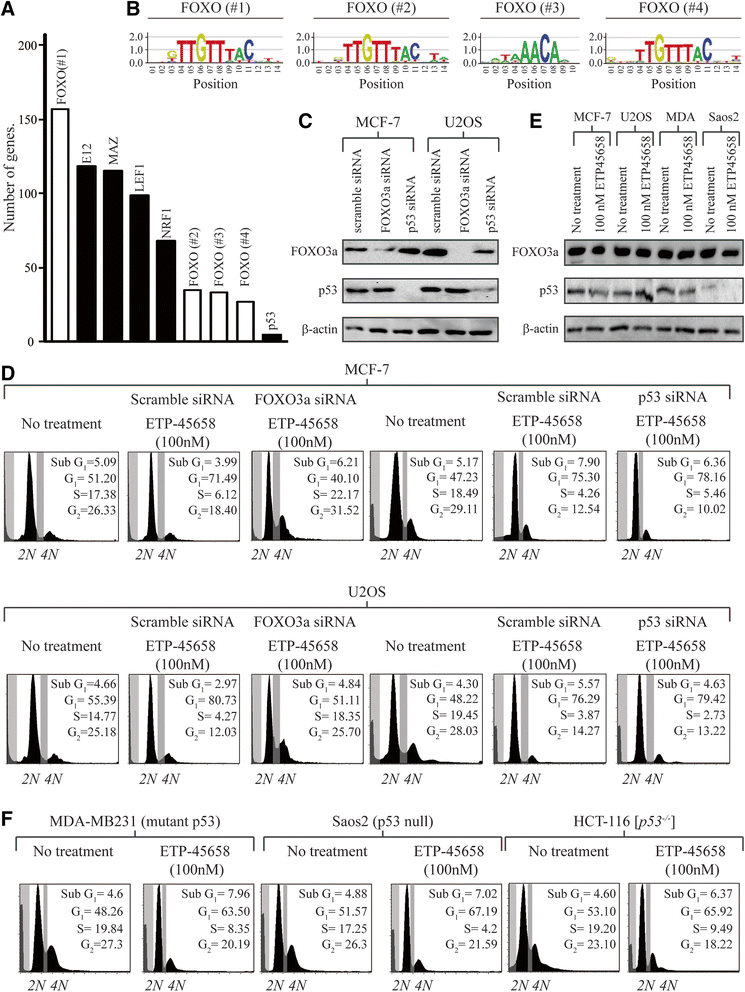

Supplement: Supplementary file 9 — Authors’ original file for figure 3 [file 13058_2014_482_MOESM9_ESM.gif]

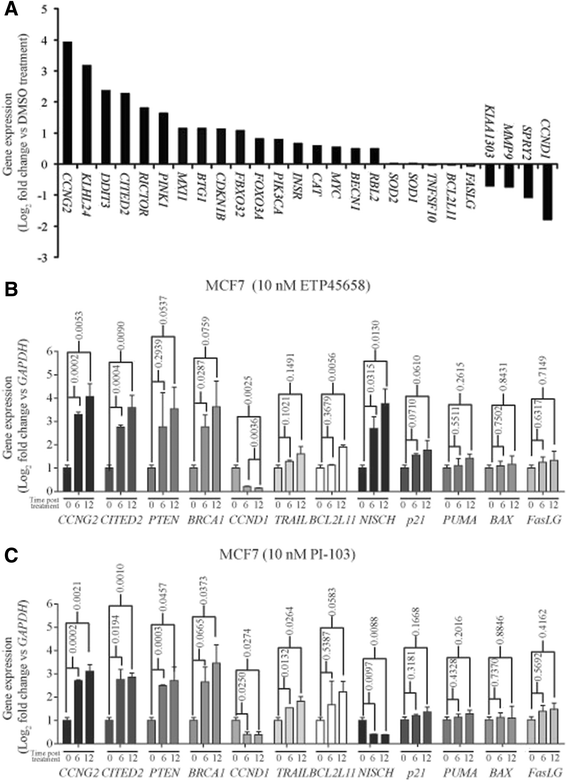

Supplement: Supplementary file 10 — Authors’ original file for figure 4 [file 13058_2014_482_MOESM10_ESM.gif]

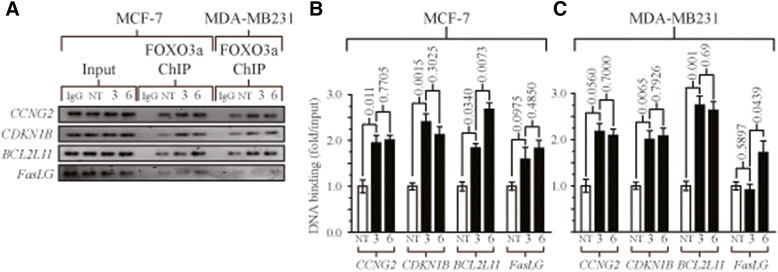

Supplement: Supplementary file 11 — Authors’ original file for figure 5 [file 13058_2014_482_MOESM11_ESM.gif]
